# Study on Flow Field Characteristics of the 90° Rectangular Elbow in the Exhaust Hood of a Uniform Push–Pull Ventilation Device

**DOI:** 10.3390/ijerph15122884

**Published:** 2018-12-16

**Authors:** Xiang Wu, Lindong Liu, Xiaowei Luo, Jianwu Chen, Jingwen Dai

**Affiliations:** 1 School of Engineering and Technology, China University of Geosciences (Beijing), Beijing 100083, China; wuxiang@cugb.edu.cn (X.W.); lld816@cugb.edu.cn (L.L.); 2 Department of Architecture and Civil Engineering, City University of Hong Kong, Hong Kong, China; 3 China Academy of Safety Science and Technology, Beijing 100029, China; cjw3000@126.com; 4 School of Water Resources and Environment, China University of Geosciences (Beijing), Beijing 100083, China; jingwen-dai@foxmail.com

**Keywords:** uniform push–pull ventilation device, 90° rectangular elbow, radius of curvature, non-uniformity, flow field characteristics

## Abstract

A uniform push–pull ventilation device can effectively improve indoor air quality (IAQ). The 90° rectangular elbow is an important part of the push–pull ventilation device. This paper analyzes the flow field characteristics of the 90° rectangular elbows under different working conditions. This was done by using computational fluid dynamics (CFD) simulation (Fluent). The flow lines, velocity and pressure distribution patterns of the elbow flow field are revealed in detail. The wind velocity non-uniformity and wind pressure non-uniformity of the 90° rectangular elbows with different coefficients of radius curvature R and rectangular section height h are also compared. The results show that when R ≥ 2.5 h, the wind flow traces inside the elbow are basically parallel lines. At the same time, the average wind velocity and the average wind pressure are stable. Also, the wind velocity non-uniformity and wind pressure non-uniformity decrease with the increase of R. Therefore, considering the space and material loss caused by an increase in radius of curvature, the elbow with R = 2.5 h can be used as the best design structure for the 90° rectangular elbow, which is of great significance for improving the control effect of dust and toxic pollutants in a uniform push–pull ventilation device.

## 1. Introduction

**Figure 1:**
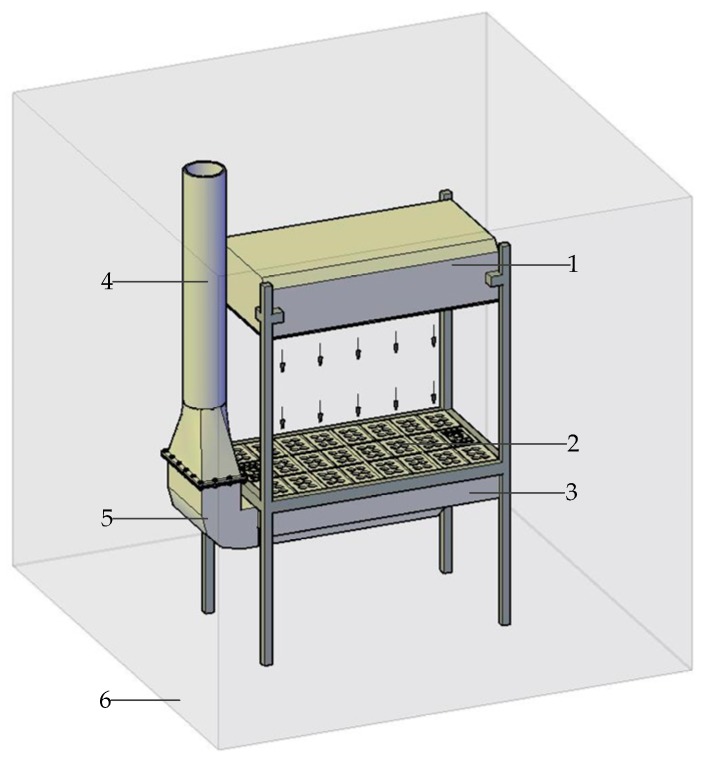
Uniform push–pull ventilation device. 1—Air-supply hood; 2—work surface; 3—exhaust hood; 4—duct; 5—90° rectangular elbow; 6—indoor workplace.

Statistics show that people spend more than 90% of their time staying in indoor environments [1]. Therefore, indoor air quality (IAQ) has an important impact on occupational health and work efficiency, which has been widely discussed [2,3,4,5]. The uniform push–pull ventilation device has a good control effect on dust and toxic pollutants, and as a result, it can effectively improve the indoor air quality [6,7]. The device is mainly used in indoor workplaces where dust and toxic pollutants may be generated, such as grinding (dust), brushing (toxic pollutants such as benzene and its derivatives), and washing electronics boards (toxic pollutants such as benzene and its derivatives). It is mainly composed of an upper air-supply hood, a lower exhaust hood and a 90° rectangular elbow (shown in Figure 1), and the wind flow is sent out by the upper air-supply hood and captured by the lower exhaust hood. The cover of the lower exhaust hood is provided with a metal orifice plate and can be used as a work surface. When the workers perform the work of generating dust and toxic pollutants on the work surface, the dust and toxic pollutants generated during the operation will be completely captured by the lower exhaust hood through the work surface under the combined action of the air-supply hood and the exhaust hood. After that, the airflow carrying the dust and toxic pollutants is discharged through the 90° rectangular elbow and the duct into the external air purifying device to prevent it from spreading to the indoor workplace. Because of the ventilation method of the upper and lower rows, the wind flow moves from top to bottom, which can effectively prevent the spread of dust and toxic pollutants in the horizontal direction, and the fresh airflow preferentially passes through the breathing belt of the workers [8,9]. It has been found in experimental studies that good control effects on dust and toxic pollutants depend on the uniformity of airflow inside the push–pull ventilation flow field of the device. The 90° rectangular elbow is an important component connecting the lower exhaust hood and the duct. The airflow carrying dust and toxic pollutants will pass through the elbow first. Thus, the elbow directly affects the airflow uniformity of the exhaust hood. Therefore, to improve the airflow uniformity of uniform push–pull ventilation device and ensure the good control effect on dust and toxic pollutants, this paper studies the flow field characteristics of the 90° rectangular elbow in the uniform push–pull ventilation device.

Berger and Smith et al. first provided extensive theoretical analysis of the elbow flow field [10,11]. However, as the flow in the elbow is much more complicated than the flow in the straight tube, it is not feasible to rely solely on experimental methods to fully investigate the characteristics of the flow field in the elbow. With the continuous development of CFD technology, Rütten et al. first used large-eddy simulations to study the flow field characteristics in the elbow. Thereafter, the numerical simulation method has been widely used in the study of the characteristics of the flow field in the elbow [12]. Combining Fluent with the experimental results, Lu et al. observed the pressure distribution of the 90° circle elbow flow field and analyzed the pressure distribution law in the separation zone, and then provided a new pressure loss calculation method [13,14,15,16]. Bohuslav Kilkovský et al. summarized several common methods for reducing the pressure loss in the 90° elbow flow field [17]. Crawford et al. studied the compound equation of the pressure loss of the 90° elbow [18]. Rup et al. experimentally analyzed the flow field characteristics of a 90° rectangular elbow, and the results showed that the fluid elements experienced large acceleration near the inner wall in the first section of the bend (0° < φ < 30°), and that the subarea near the outer wall of the bend had local vortices [19]. Röhrig et al. observed the 90° elbow flow field when comparing the large-eddy simulation (LES) and Reynolds-averaged Navier–Stokes (RNS) methods, and the results showed that the mean velocity field of the flow through a pipe elbow can be predicted accurately, and that occurring vortices can be also captured precisely [20]. Kim et al. experimentally and numerically studied the pressure variation in the flow field of 90° elbows and fillet elbows, and they found that there are significant pressure changes in the 90° elbow, while the rounded elbow has a relatively small pressure change. By comparing the experimental results and simulative results, the validity of the CFD simulation was further verified [21]. Gao et al. investigated the internal resistance of the convection field with different curvatures of a 90° rectangular elbow through numerical simulations, and found that the optimized elbow can reduce the resistance by 9–10% [22].

The above studies mainly focused on the flow field characteristics of a 90° round elbow, but studies about the 90° rectangular elbows are limited. Although round elbows are better than rectangular elbows in terms of engineering parameters, rectangular elbows have lower technical requirements than round elbows and are still widely used in ventilation system design, especially in the push–pull ventilation device. The structure of the 90° rectangular elbow is more complex than that of the round elbow. Therefore, in order to improve the uniformity of airflow inside the uniform push–pull ventilation device, it is necessary to study the flow field characteristics of the 90° rectangular elbow. This paper studies the flow field characteristics of the 90° rectangular elbows under different working conditions. The flow lines, velocity and pressure distribution patterns of the elbow flow field are analyzed. Based on a non-uniformity method, the wind velocity non-uniformity and wind pressure non-uniformity of the 90° rectangular elbows with different coefficients of radius curvature R and rectangular section height h are also compared, and the optimal design structure of the 90° rectangular elbows is suggested.

## 2. Materials and Methods

### 2.1. Research Condition

Fluent, as an advanced and widely used CFD software, was used to solve the model calculation in this study. For the typical working conditions of the 90° elbow, CAD was used to draw the geometric modeling, see Figure 1, where h is the height of the elbow rectangular section, and R is the radius of curvature of the centerline of the rectangular elbow. The inner (small) and outer (large) wall surfaces of the elbow are concentric arcs. The wide use of industrial ventilation equipment provides the design criteria for a common round bend [23]. In order to investigate the wind flow traces, pressure distribution and velocity distribution in the rectangular elbows, six types of 90° rectangular duct elbows were set up in accordance with the design criteria given by the round elbows. The elbows with a right-angled inner wall (recorded as R = 0 h) and a radius of curvature of R = 1 h, R = 2 h, R = 2.5 h, R = 3 h and R = 4 h are analyzed, respectively.

### 2.2. Model Establishment

**Figure 2:**
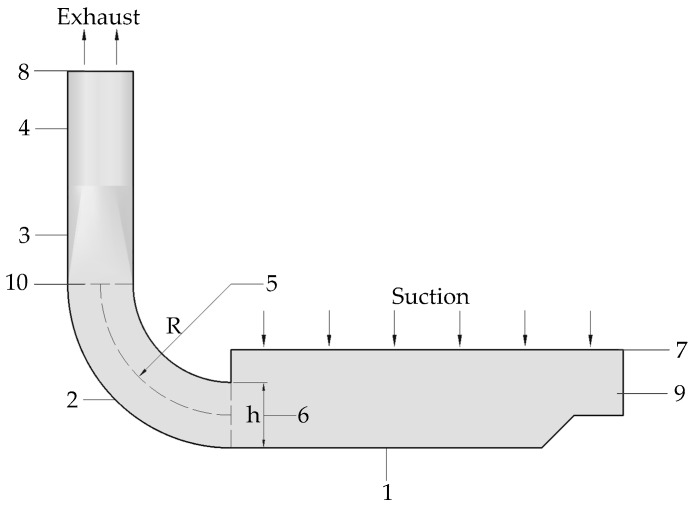
Model components and positional relationship between R and h. 1—Inlet hood; 2—rectangular elbow; 3—reducer; 4—duct; 5—radius of curvature R; 6—rectangular section height h; 7—suction boundary; 8—exhaust boundary; 9—XOZ section; 10—XOY section.

The 90° rectangular elbow is located in the push–pull ventilation exhaust hood unit. An exhaust hood device model containing elbows was established. The suction is a rectangular air inlet with dimensions of 1200 mm × 650 mm, and the exhaust is a round duct with diameter of 200 mm. The exhaust pipe uses a round pipe and the 90° rectangular elbow has a high cross-section height of h = 200 mm, and 410 mm in width. Respectively, the designs of the R = 0 h, R = 1 h, R = 2 h, R = 2.5 h, R = 3 h and R = 4 h elbow models are established. The relationship between the components of the model and R and h is shown in Figure 2. 

### 2.3. Mesh

A simplified model of the computational ventilation system was introduced into Gambit’s software. Tetragonal hybrid mesh (Tet/Hybrid) was chosen, where the tetrahedral meshes are dominant and the hexahedral meshes are used in some areas. The grid is divided by 0.5 according to the grid independence test. In order to prevent the elbow part in the vicinity of the boundary layer of the grid from being automatically smoothed and then causing unnecessary errors, the AUTO_SMOOTH option was set to 0 to turn off the automatic smoothing grid.

### 2.4. Control Equations, Boundary Conditions and Solution Parameters

The numerical calculation uses a single-precision 3D solver and a standard k-ε double-equation model [24]. The model is a semi-empirical model. It is assumed that the gas in the flow field is a Newtonian and incompressible fluid. It is a fully developed turbulent flow and the viscosity between fluid molecules is negligible. Overall, the standard *k*-*ε* double-equation model has a reasonable accuracy and it has been widely used in the engineering applications. Only the momentum transmission is considered in the model and heat transfer is ignored. The specific form of the *k*-*ε* double-equation model is shown in Equations (1) and (2).

*k* equation:

(1) $$\frac{\partial }{\partial x_{i}}\left(\rho u_{i}k\right)=\frac{\partial }{\partial x_{i}}\left[\left(\mu +\frac{\mu_{t}}{\sigma_{k}}\right)\frac{\partial k}{\partial x_{i}}\right]+G_{k}-\rho \varepsilon ,$$

*ε* equation:

(2) $$\frac{\partial }{\partial x_{i}}\left(\rho u_{i}\varepsilon \right)=\frac{\partial }{\partial x_{i}}\left[\left(\mu +\frac{\mu_{t}}{\sigma_{k}}\right)\frac{\partial k}{\partial x_{i}}\right]+\frac{C_{\varepsilon 1}\varepsilon }{k}G_{k}-C_{\varepsilon 2}\varepsilon \rho \frac{\varepsilon^{2}}{k}.$$

The boundary condition of the wind inlet in the elbow model was set to velocity-inlet. The suction area is 0.78 m^2^ and the exhaust area is 0.1256 m^2^. The wind velocity of suction is 2.1 m/s, and the wind velocity of exhaust is defined by Equation (3) [23]:

(3) $$v_{suction}\times s_{suction}=v_{exhaust}\times s_{exhaust}.$$

The wind velocity of exhaust is found to be 13 m/s. The ventilation model inlet diameter is 0.2 m and the turbulence intensity is 3.62%; the model outlet boundary condition is pressure outlet, the hydraulic diameter is 0.84 m, and the turbulent intensity is 3.03%. In order to discretize the computational domain, the rest of the model was set to the Discrete Particle Model (DPM) condition except for the inlet and outlet of the airflow. The suction and exhaust boundary condition is shown in Figure 2. These parameters are shown in Table 1.

The study uses the SIMPLEC algorithm as solver, which is one of the most widely used flow field calculation methods in engineering. It is one of the pressure correction methods. This method was proposed by Patankar and Spalding in 1972, and it is a numerical method for solving incompressible flow fields [25]. The core is to use the “guess-correction” process to calculate the pressure field on the basis of a staggered grid, which can be used to solve the Navier–Strokes equation. The solver parameters set here are shown in Table 2.

## 3. Results

### 3.1. Influence of Curvature Radius on Wind Trace

**Figure 3:**
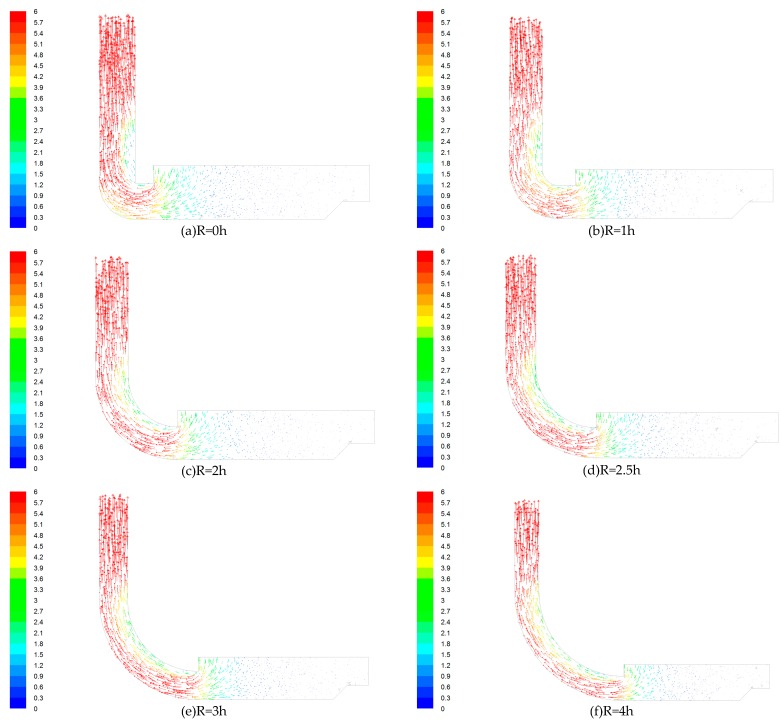
Wind flow traces. (**a**) R = 0 h; (**b**) R = 1 h; (**c**) R = 2 h; (**d**) R = 2.5 h; (**e**) R = 3 h; (**f**) R = 4 h.

The air flow is sucked by the suction and exhausted by the exhaust. Figure 3 shows the wind flow traces in a 90° rectangular elbow with different radii of curvature on the XOZ section shown in Figure 2. The different colors in the left colormap correspond to different wind velocities on the traces.

As can be seen from Figure 3, when R = 0 h, there is a clear vortex close to the inner wall surface. The vortex close to the inner wall surface of the elbow with a radius of curvature R = 1 h is significantly reduced when compared to the R = 0 h elbow, and the wind trace opposite to the main flow direction disappeared. When the curvature radius changed from R = 1 h to R = 4 h, the wind flows opposite to the mainstream wind traces continued to reduce until they disappeared, and the final wind traces were basically parallel lines. Wang et al. studied the instantaneous turbulent flow field of a 90° elbow and their results are similar to the results shown in Figure 3 [26]. In some cases, because there are a large number of opposite wind traces in the vortex, which are inconsistent with the main flow direction, the ventilation effect is significantly reduced.

### 3.2. Ventilation Non-Uniformity

**Figure 4:**
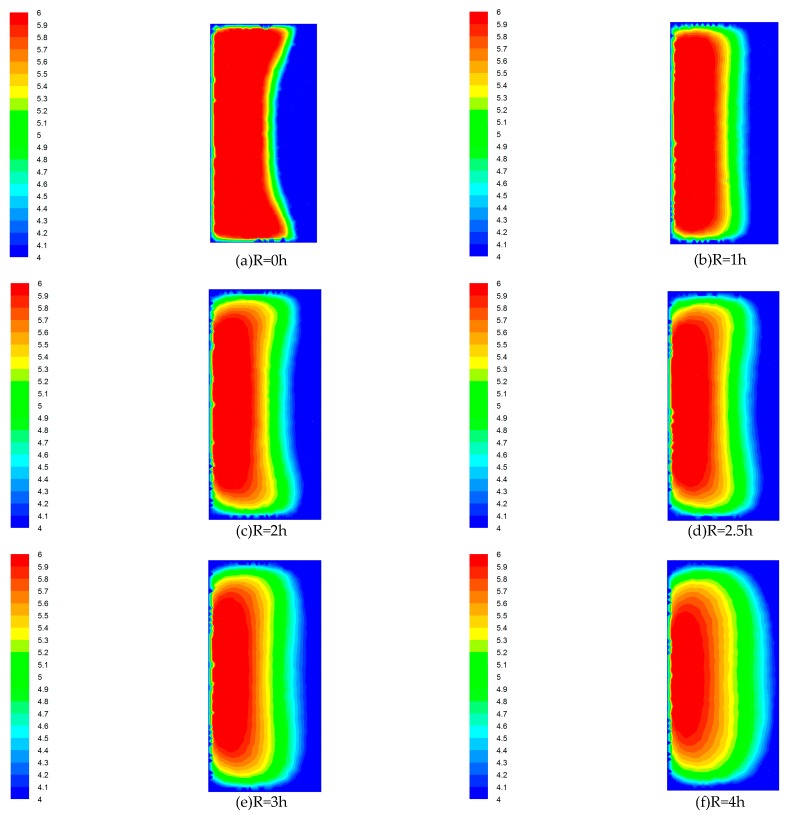
Velocity distribution cloud. (**a**) R = 0 h; (**b**) R = 1 h; (**c**) R = 2 h; (**d**) R = 2.5 h; (**e**) R = 3 h; (**f**) R = 4 h.

In order to further study the characteristics of wind flow in the flow field, the distribution of wind velocity in the elbow exit section (XOY section shown in Figure 2) at the outlet of the 90° rectangular elbow was studied. The velocity distribution of a 90° rectangular elbow with different radii of curvature is shown in Figure 4. The different colors on the left side colormap correspond to different wind velocities in the cloud.

As shown in Figure 4, in the elbow exit section with R = 0 h, the wind velocity close to the inner wall surface is low, and the wind velocity close to the outer wall surface is relatively large. The distribution of wind velocity is extremely uneven. The wind velocity uniformity at the elbow with R = 1 h is significantly improved when compared to the elbow with R = 0 h, but the wind velocity close to the inner wall surface is still much smaller than the wind velocity close to the outer wall surface. From R = 1 h to R = 4 h elbow, the wind velocity difference became smaller and smaller, and the section wind velocity uniformity was obviously improved.

Nine evenly distributed measuring points were set to calculate the average velocity. The calculation results of the average wind velocity in the elbow exit section are given in Table 3. The formula for calculating the wind velocity non-uniformity is shown in Equation (4).

(4) $$\beta =\frac{\sqrt{\frac{\sum {\left(v_{i}-\bar{v}\right)}^{2}}{n-1}}}{\bar{v}},$$

where:

*β*: uniformity of wind velocity;
*v_i_*: wind velocity at any measurement point;
$\bar{v}$: average wind velocity; and
*n*: number of measuring points.

It can be known from Table 3 that the average wind velocity continues to decrease from R = 0 h to R = 2.5 h, and then increases from R = 2.5 h to R = 4 h, and meanwhile, the wind velocity non-uniformity continues to decrease.

### 3.3. Bend Pressure Loss

Wind pressure is also an important indicator for evaluating the characteristics of the flow field in the elbow.

**Figure 5:**
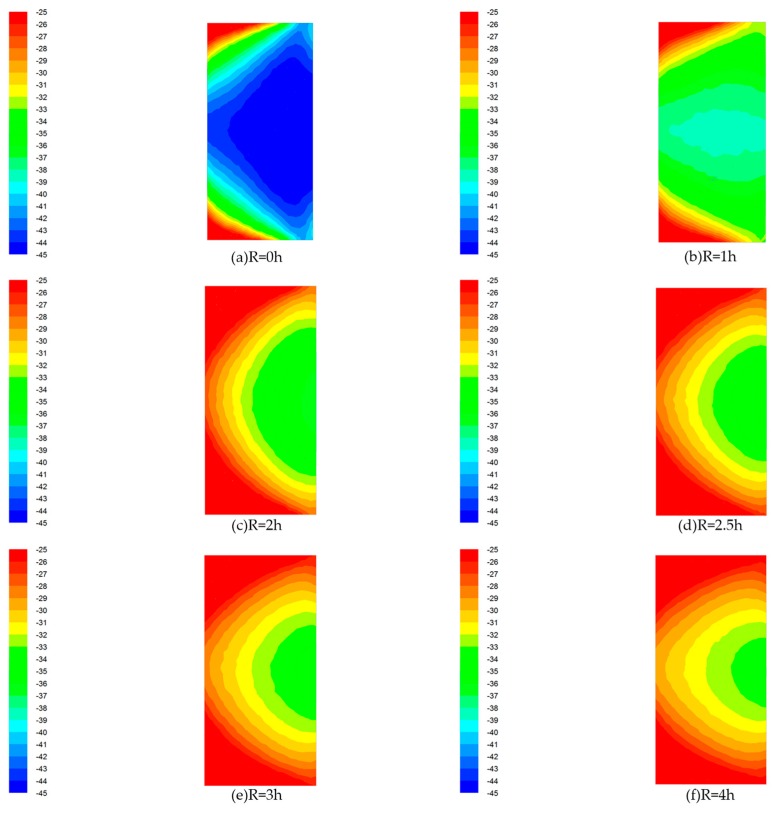
Pressure distribution cloud. (**a**) R = 0 h; (**b**) R = 1 h; (**c**) R = 2 h; (**d**) R = 2.5 h; (**e**) R = 3 h; (**f**) R = 4 h.

Figure 5 shows the pressure distribution in the elbow exit section (XOY section shown in Figure 2) with different radii of curvature. The different colors in the left colormap correspond to different wind pressures.

Figure 5 shows that there is a big difference of wind pressure in the elbow exit section with R = 0 h. The wind pressure at the two corners of the outer wall is larger and the wind pressure distribution is extremely uneven, and therefore the pressure gradient is extremely large. The wind pressure uniformity at the R = 1 h elbow is significantly improved compared with the elbow with R = 0 h, but there is still a significant pressure gradient close to the corner of the outer wall. From the R = 1 h to R = 4 h elbow, the pressure gradient of the elbow exit section wind pressure became smaller and smaller, and the uniformity of the elbow exit section wind pressure was obviously improved.

Nine evenly distributed measuring points were set to calculate the average pressure. The calculation results of the average wind pressure in the elbow exit section are given in Table 4.

The data in Table 4 shows that the average wind pressure continues to decrease from R = 0 h to R = 4 h, but the change from the R = 2 h elbow is no longer obvious, and the wind pressure non-uniformity continues to decrease from R = 2 h.

## 4. Discussion

Through the study of the wind flow lines, it was found that there is a subflow opposite to the trace of the mainstream wind flow trace; there is a clear vortex close to the inner wall surface, the velocity of the airflow near the inner surface of the elbow is lower, and the velocity of the airflow near the outer surface of the elbow is higher. Rup et al. also found the same phenomenon when studying the flow field characteristics of right-angle elbows, and observed the difference in velocity and eddy current between the inner and outer walls [19]. After changing the radius of curvature of the elbow, it can be seen from Figure 3 that the elbow with R = 1 h has a significantly small vortex close to the inner wall surface compared with that in the R = 0 h elbow, and the wind flow trace that is opposite to the main flow direction basically disappears. When the elbow with a larger radius of curvature is used (R changes from 1 h to 4 h), it can be observed that the flow in the elbow that is opposite to the trace of the mainstream continues to decrease until it disappears, and finally the wind flow trace is substantially parallel. This indicates that the use of elbows with a large radius of curvature can significantly reduce the movement of the internal wind flow opposite to the main flow, and then avoid unnecessary energy exchange. According to the above findings, it can be preliminarily concluded that the influence of the elbow with larger radius of curvature on the airflow conveying process is smaller, especially when R ≥ 2.5 h.

Through the study of the average wind velocity and wind velocity non-uniformity, Table 3 shows that the average wind velocity continues to decrease from R = 0 h to R = 2.5 h, and then increases from R = 2.5 h to R = 4 h. Therefore, the elbow with R = 2.5 h is the elbow whose average wind velocity starts to increase. Moreover, from the R = 0 h to R = 4 h elbow, the uniformity of airflow is also significantly improved. Table 3 shows that the airflow non-uniformity decreases from 48.84% to 14.98%, and the uniformity of the airflow increased by 33.86%. It is proved that the use of elbows with radius of curvature has a significant effect on improving the uniformity of wind flow in the elbow. With the increase of the radius of curvature, the uniformity of the airflow in the elbow is more obvious. Combined with the average wind velocity parameter, it is concluded that when the elbow with R ≥ 2.5 h is used, the airflow movement in the elbow is less affected, and the airflow non-uniformity at the elbow can be reduced by about 30%. Therefore, in order to improve the uniformity of the airflow in the uniform push–pull ventilation device, the 90° rectangular elbow with a radius of curvature R ≥ 2.5 h should be used.

Through the study of the average wind pressure and pressure non-uniformity, Table 4 shows that in the elbow with radius of curvature changing from R = 0 h to R = 2 h, the loss of the average wind pressure decreases significantly with the increase of R, the average wind pressure increased from −40.92 Pa to −29.31 Pa, and the loss of the average wind pressure decreased by 30.13%, but there was a significant pressure gradient in the elbow; the pressure loss in the elbow with R = 2 h to R = 4 h does not changes significantly with the increase of R, and the pressure gradient in the elbow is significantly ameliorated; when R ≥ 2.5 h, the pressure loss slightly decreases and the pressure gradient is significantly reduced. Gao et al. found that the optimized elbow can reduce the resistance by 9–10% [22], while this study found that it can reduce it by 30.13%. Lower pressure loss means better control of dust and toxic pollutants. Furthermore, in Figure 5, it can be seen that there is a large radial pressure gradient inside the elbow when R = 0 h. The pressure gradient between the inner wall surface and the outer wall surface of the 90° rectangular elbow is obviously different. Specifically, the pressure in the vicinity of the inner wall surface is small, and the pressure in the vicinity of the outer wall surface is large. However, compared with the R = 0 h elbow, the pressure gradient of elbow with R = 1 h to R = 4 h is significantly reduced, which has a significant effect on improving the uniformity in the elbow. Kim et al. studied the characteristics of the flow field inside in the right-angle elbow and the fillet elbow flow field, and found that the right-angle elbow has a significant pressure gradient [21], while the fillet elbow has a smaller pressure gradient. This is consistent with the conclusions of this paper.

The simulation results show that the larger the radius of curvature, the more uniform the flow field in the rectangular elbow after R ≥ 2.5 h. However, in the actual engineering design, considering the space and material loss caused by the increase of the radius of curvature, the radius of curvature R = 2.5 h can be considered as the best design structure for the 90° rectangular elbow. When the elbow of R = 2.5 h is used, the uniformity of the average wind velocity and the average wind pressure in the 90° rectangular elbow can be ensured under the condition of avoiding greater pressure loss, and the stability of the air flow transmission process can be effectively improved. Under this condition, good airflow uniformity in the 90° rectangular elbow will improve the airflow uniformity of the exhaust hood, which is important for the uniformity of the airflow in the push–pull ventilation flow field.

## 5. Conclusions

Through the simulation calculations of 90° rectangular elbows under different working conditions, the flow field characteristics of 90° rectangular elbows with six different coefficients were analyzed. From the study of the wind traces, pressure and velocity distribution, and wind velocity and wind pressure non-uniformity, it can be concluded that when R ≥ 2.5 h, the wind flow traces in the elbow are basically parallel lines. At the same time, when the average wind velocity and the average wind pressure are stable, the wind velocity non-uniformity and wind pressure non-uniformity decrease with the increase of R, which is reduced by 33.86% and 30.13%, respectively. Therefore, combined with actual engineering design, the elbow with R = 2.5 h can be used as the best design structure for the 90° rectangular elbow. At this time, the uniformity of the airflow in the push–pull ventilation flow field can be effectively improved, which can better control dust and toxic pollutants.

## Figures and Tables

**Table 1:** Boundary conditions.

Boundary Conditions	Definition
Exhaust boundary type	Velocity-inlet
Inlet velocity magnitude (m/s)	−13
Hydraulic diameter (m)	0.2
Turbulence intensity (%)	3.62
Suction boundary type	Pressure outlet
Hydraulic diameter (m)	0.84
Turbulence intensity (%)	3.03

**Table 2:** Solver parameter settings.

Solver	Definition
Solver	Segregated
Viscous model	*k*-epsilon (*k*-*ε*)
Pressure–velocity coupling	SIMPLEC
Discretization scheme	Second-order upwind
Convergence criterion	10^−6^

**Table 3:** Wind velocity distribution and non-uniformity.

Elbow Type	Average Wind Velocity (m/s)	Non-Uniformity (%)
R = 0 h	5.10	48.84
R = 1 h	4.96	29.67
R = 2 h	4.93	22.13
R = 2.5 h	4.88	20.65
R = 3 h	4.91	18.47
R = 4 h	4.91	14.98

**Table 4:** Wind pressure distribution and non-uniformity.

Elbow Type	Average Wind Pressure (Pa)	Non-Uniformity (%)
R = 0 h	−40.92	9.69
R = 1 h	−34.58	8.00
R = 2 h	−29.31	14.40
R = 2.5 h	−28.80	12.79
R = 3 h	−28.60	11.64
R = 4 h	−28.59	10.24
